# Low‐Frequency Noise Spectroscopy for Navigating Geometrically Varying Strain Effects in HfO_2_ Ferroelectric FETs

**DOI:** 10.1002/advs.202501367

**Published:** 2025-04-02

**Authors:** Ryun‐Han Koo, Wonjun Shin, Sangwoo Kim, Jangsaeng Kim, Been Kwak, Jiseong Im, Hyunwoo Kim, Deok‐Hwang Kwon, Suraj S. Cheema, Jong‐Ho Lee, Daewoong Kwon

**Affiliations:** ^1^ Department of Electrical and Computer Engineering and Inter‐university Semiconductor Research Center Seoul National University Seoul 08826 Republic of Korea; ^2^ Department of Semiconductor Convergence Engineering Sungkyunkwan University Suwon 16419 Republic of Korea; ^3^ Research Laboratory of Electronics Massachusetts Institute of Technology Cambridge MA 01239 USA; ^4^ Department of Electrical Engineering Hanyang University Seoul 04763 Republic of Korea; ^5^ Department of Electronic Engineering Department of System Semiconductor Engineering Sogang University Seoul 04107 Republic of Korea; ^6^ Department of Electrical and Electronics Engineering Konkuk University Seoul 27478 Republic of Korea; ^7^ Center for Energy Materials Research Korea Institute of Science and Technology Seoul 02792 Republic of Korea

**Keywords:** HfO_2_ ferroelectric thin films, low‐frequency noise, poly‐Si, strain

## Abstract

Strain engineering has been widely employed to control and enhance the ferroelectric properties of hafnium oxide (HfO₂)‐based thin films. While previous studies focused on the influence of the strain in simple metal‐ferroelectric‐metal structures, the integration of strain‐induced ferroelectricity into field‐effect transistors (FETs) requires consideration of geometrical factors, such as the interfaces between the channel and source/drain contacts, as well as device dimension. Here, we demonstrate strain effects in HfO₂‐based ferroelectric FETs (FeFETs) with poly‐Si channels via low‐frequency noise (LFN) spectroscopy. LFN analysis reveals that the strain during the post‐metal annealing introduces damage to channel interface with its severity depending on the device geometry. This strain‐dependent behavior results in a unique noise characteristic, which we refer to as the *reverse scaling effect*, where noise increases with longer channel lengths—contrary to the conventional trend in typical FETs, where noise decreases with increasing channel length. Furthermore, we observe that while increased strain enhances ferroelectricity, it also degrades the electrical performance of poly‐Si FeFETs, primarily through damage to the channel interfaces. These findings underscore the critical role of strain engineering in FeFETs and provide important guidelines for balancing strain effects to achieve optimal ferroelectricity and reliability in future device designs.

## Graphical abstract

This study investigates the material and geometrically varying strain effects on HfO_2_ ferroelectric FETs (FeFETs). Through noise analysis, the reverse scaling effect is demonstrated due to the strain effects in the FeFET structure. This result indicates the importance of strain engineering in the FeFET for optimized performance.

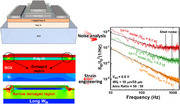

## 1 Introduction

Fluorite‐structure ferroelectric (FF) thin films, such as doped HfO_2_ and ZrO_2_, have attracted considerable attention in material science and electronic devices.^[^ ^1^, ^2^, ^3^ ^]^ These films have a wide range of potential applications, such as non‐volatile memory, chemical sensing, and neuromorphic computing.^[^ ^4^, ^5^, ^6^ ^]^ Their compatibility with complementary metal‐oxide‐semiconductor (CMOS) technology makes them particularly attractive for integration into existing electronic systems. A key advantage of FF films is their CMOS compatibility, high scalability (sub 10 nm), and fast polarization switching capabilities, which make them promising candidates for next‐generation devices, potentially replacing conventional charge trap‐based memories.^[^ ^7^ ^]^ In addition, the ability of ferroelectric (FE) materials to achieve partial polarization allows for multilevel conductance. This feature is highly advantageous in neuromorphic computing applications that rely on different conductance states for mimicking long‐term potentiation and depression. Consequently, significant research efforts have been dedicated to improving the ferroelectric properties of FFs to enhance the device performance and unlock their full potential.^[^ ^8^, ^9^, ^10^ ^]^

The ferroelectric properties of FFs are significantly influenced by the stress/strain state of the films.^[^ ^11^, ^12^, ^13^ ^]^ These properties are primarily governed by the displacement of ions within the sub‐unit cell, particularly when the crystal structure supports multiple stable positions for certain ions.^[^ ^11^ ^]^ In the case of HfO_2_, the ferroelectric behavior is induced by the appearance of a metastable polar orthorhombic phase (o‐phase) or a rhombohedral phase (r‐phase), along with nonpolar phases such as monoclinic phase (m‐phase) or tetragonal phase (t‐phase).^[^ ^14^ ^]^ The presence of strain can promote or suppress the formation of these phases, thereby influencing the ferroelectric behavior. The strain also has a significant impact on the energy landscape and energy barriers associated with polarization switching in ferroelectric materials. This influence extends to parameters such as the coercive field, which represents the electric field required to switch the polarization, and the remnant polarization, which denotes the polarization that remains after the electric field is removed.^[^ ^15^ ^]^ Consequently, extensive research has been carried out to investigate the effects of strain on the ferroelectricity of HfO_2_. Several methods have been proposed to engineer and control these strain effects.^[^ ^16^, ^17^, ^18^, ^19^, ^20^ ^]^ One common approach is the post‐metal annealing process, which induces mechanical strain, including both compressive and tensile stresses, in ferroelectric materials by exploiting the differences in their coefficients of thermal expansion (CTE).^[^ ^16^ ^]^ The strain in ferroelectric materials can be controlled by carefully selecting and engineering the configuration or types of materials used in the top and bottom electrodes or substrate.^[^ ^17^ ^]^ Consequently, significant research efforts have been dedicated to enhancing ferroelectricity by optimizing the CTE between the materials that compose ferroelectric devices. Previous studies have utilized metal‐ferroelectric‐metal (MFM) capacitors or ferroelectric tunnel junctions (FTJs) to examine the effects of mechanical strain on ferroelectricity.^[^ ^16^, ^17^, ^18^, ^19^, ^20^ ^]^ The adoption of FTJs is primarily due to their simplified structure, which facilitates the analysis of the influence of mechanical strain on ferroelectric behavior (**Table** **1**).

**Table 1:** Summary of previous study on strain engineering of HfO_2_‐based ferroelectric devices.

Year	Device structure [Bottom to top electrode]	Device Configuration	Strain Engineering	Ferroelectric thickness	2Pr [µC/cm^2^]	Refs.
2011	TiN/Si:HfO_2_/TiN	Two‐terminal MFM	Si doping	10.0	22.4	[11]
2012	TiN/HZO/TiN	Two‐terminal MFM	Zr doping	9.0	57.6	[12]
2013	TiN/HZO/TiN	Two‐terminal MFIS	Strain Mismatch	‐	‐	[19]
2020	Si/SiO_2_/HZO/TiN	Two‐terminal MFIS	Zr doping	Sub‐2	‐	[13]
2020	TiN/HZO/RuO_2_/T	Two‐terminal MFIM	Interlayer Insertion	10.0	54.8	[15]
2020	W/HZO/TiN	Two‐terminal MFM	Bottom electrode	4.5	62.4	[16]
2020	TiN/TiO_2_/HZO /RuO_2_	Two‐terminal MFM	High‐pressure annealing	8.0	33.6	[17]
2021	TiN/HZO/TiO_2_/ HZO/TiN	Two‐terminal MFIM	Interlayer insertion	22/10	32.4	[14]
2021	TiN/TiO_2_/HZO/ HfO_2_/TiN	Two‐terminal MFIM	Interlayer insertion	10.0	33.2	[18]
2021	SiO_2_/HfO_2_/TiN	Two‐terminal MFIS	Post‐metal Annealing	‐	‐	[20]
2025	Poly‐Si/ZrO_2_/ Mo/HZO/Mo	Three‐terminal FeFET	Interlayer insertion/ Geometrical variation	8.0	51.5	This Work

However, the successful integration of ferroelectricity into memory or neuromorphic applications necessitates the development of ferroelectric field‐effect transistors (FeFETs), which offer superior scalability and reliability compared with FTJs.^[^ ^21^, ^22^, ^23^ ^]^ In Particular, in the context of 3D vertical NAND (VNAND) technology, where the potential to replace conventional charge trap‐based memories with ferroelectricity is highly promising,^[^ ^24^, ^25^ ^]^ it becomes crucial to investigate the effects of strain on FETs. Given that commercial VNAND utilizes poly‐silicon (poly‐Si), the effect of mechanical strain on FETs with a poly‐Si channel requires thorough studies. It is important to note that poly‐Si is more strongly affected by mechanical strain than single crystalline Si.^[^ ^26^, ^27^ ^]^ Therefore, comprehensive studies on the strain effects should encompass investigations of the influences of both ferroelectricity and poly‐Si channels. Furthermore, it is important to note that strain effects are influenced not only by material characteristics, such as the CTE, but also by the geometrical parameters of the FETs.^[^ ^28^ ^]^ Therefore, comprehensive studies that consider both the materials and geometrical effects are necessary for understanding the effect of strain on device performance. By considering these factors collectively, a better understanding of the strain effects can be gained.

This study focuses on examining the strain effects on metal‐ferroelectric‐metal‐insulator‐semiconductor (MFMIS) structure FeFETs with a poly‐Si channel based on ferroelectric hafnium‐zirconium oxide (HZO). Recent studies have highlighted the potential of MFMIS FeFETs with poly‐Si channels as promising candidates for 3D VNAND cell structure.^[^ ^25^ ^]^ Moreover, the utilization of the MFMIS structure enables us to specifically examine the effects of various geometrical factors on the electrical properties of FeFETs. In this study, both the material and geometrical effects of strain on FeFETs are comprehensively investigated. First, different insulator layers (IL) are employed to explore the materials‐related effects of strain. Subsequently, we delve into the investigation of strain effects caused by geometric factors, including the length and width of the metal beams used in the FET construction, as well as the influence of the edges. Conventional electrical measurements, such as the positive‐up‐negative‐down (PUND) measurement, pose challenges in analyzing the ferroelectricity in FeFETs with poly‐Si channels owing to trapping effects. Hence, this study employs low‐frequency noise (LFN) spectroscopy as an alternative technique. LFN spectroscopy provides valuable insights into the electrical characteristics of electronic devices with exceptional sensitivity to defects.^[^ ^29^, ^30^ ^]^ Its non‐destructive nature enables repeated monitoring of electrical characteristics under different conditions, facilitating a comprehensive understanding of device performance. Unlike the conventional trend in typical FETs, where noise decreases with increasing channel length, a phenomenon referred to as the *reverse scaling effect* has been identified in MFMIS FeFETs, where noise increases with longer channels due to strain stress in the top metal. The underlying cause of this effect has been thoroughly analyzed. The results demonstrate that both material and geometrical factors significantly influence strain effects and the overall electrical properties of MFMIS FeFETs, beyond what can be explained by conventional capacitance‐based interpretations. Technology computer‐aided design (TCAD) simulations provide further support for these findings. By integrating materials analysis, electrical measurements, and TCAD simulations, this study advances our understanding of the effects of strain on FeFETs. Further, it sheds light on the interplay between material and geometrical factors and their influence on the performance of these devices, which is valuable for the development of improved FeFET‐based technologies.

## 2 Results and Discussion

### 2.1 Ferroelectricity of the HZO Film

**Figure 1:**
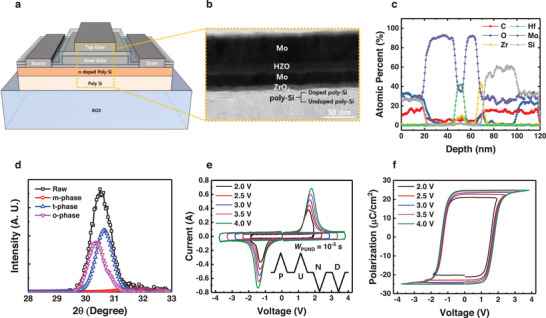
a) 3D bird's eye view of the FeFET with MFMIS structure. b) Cross‐sectional TEM image of the MFMIS (Mo (top metal) /HZO (ferroelectric layer)/ Mo (inner metal) / ZrO_2_ (IL)/ poly‐Si (semiconductor channel)) stack incorporated into the device. c) Energy‐ dispersive X‐ray spectroscopy (EDS) line scan of the corresponding stack d) X‐ray diffraction analysis (XRD) of the HZO layer. e) Bias scheme for PUND measurement. f) Polarization versus frequency of the ferroelectric HZO layer.

In this study, we adopt HZO as a ferroelectric material that exhibits polarization at low temperatures. **Figure** **1a** shows a 3D bird's eye view of an FeFET with an MFMIS structure. The fabrication process of the devices is described in Figure S1 (Supporting Information) and Method section. Figure 1b shows the cross‐sectional transmission electron microscopy (TEM) image of the MFMIS (Mo (top metal) /HZO (ferroelectric layer)/ Mo (inner metal) / ZrO_2_ (IL)/ poly‐Si (semiconductor channel)) stack incorporated into the device. Figure 1c shows an energy‐dispersive X‐ray spectroscopy (EDS) line scan of the corresponding stack. In order to examine the ferroelectricity of HZO, the X‐ray diffraction analysis (XRD) is conducted, as shown in Figure 1d. HZO exhibits t‐, o‐, and m‐phases. It has been reported that the o‐phase induces ferroelectricity of the HZO stack. To investigate the polarization properties of HZO, PUND measurements are conducted with a pulse width of 10^−5^ s, and are shown in Figure 1e. Figure 1f shows the polarization versus voltage curves of the HZO measured at different bias sweep ranges. The remnant polarization of the HZO is +24.9 and −25.1 µC cm^2^ in the bias sweep ranges between −4 and +4 V, demonstrating excellent ferroelectricity.

### 2.2 Electrical Characteristics of the MFMIS FeFETs

#### 2.2.1 Effects of IL Material on Tensile Strain

**Figure 2:**
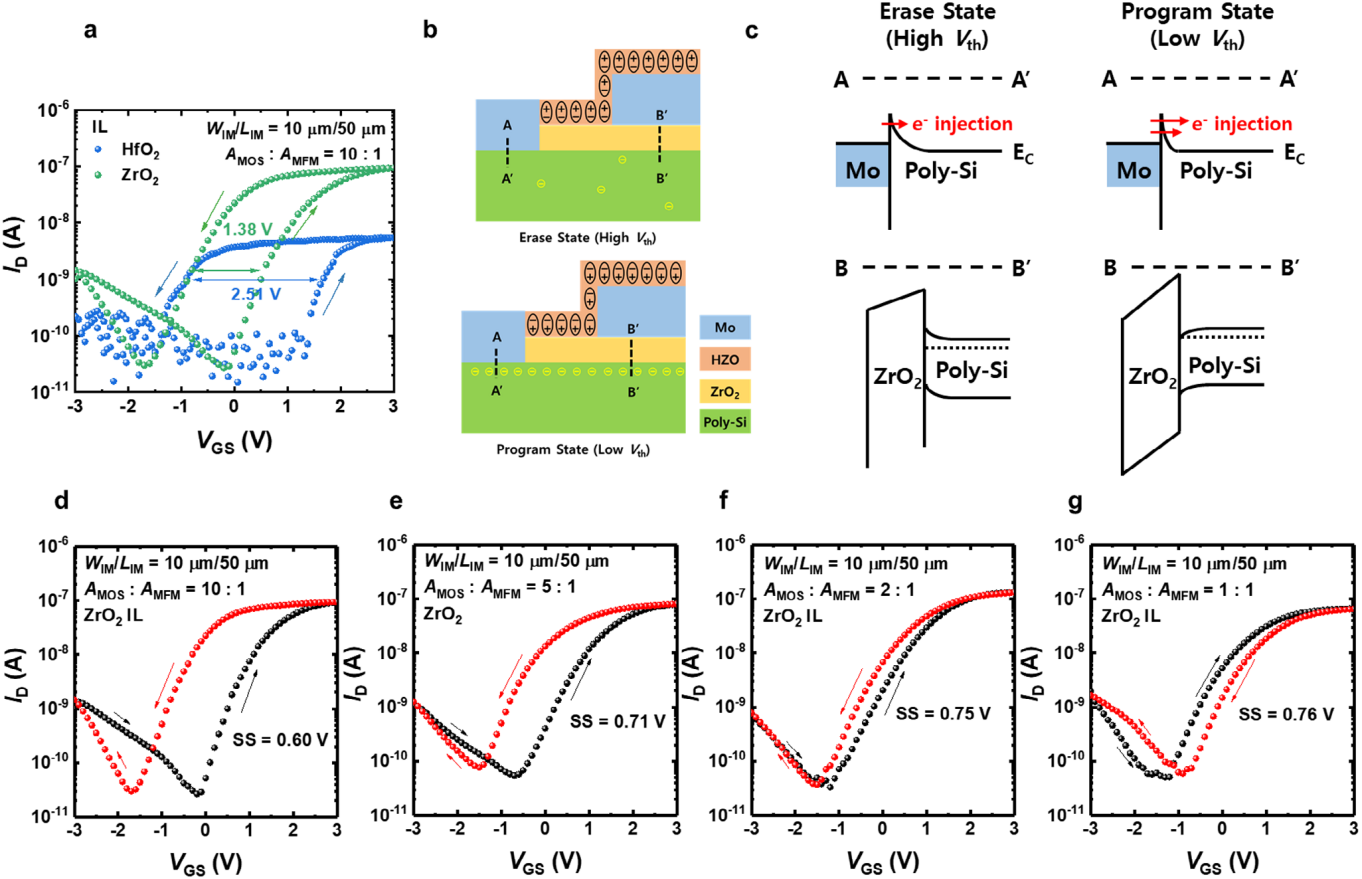
a) Double‐sweep transfer characteristics (*I*_D_‐*V*_GS_) of the MFMIS FeFETs with ZrO_2_ and HfO_2_ ILs, denoted by green and blue symbols, respectively. The inner metal has a width (*W*_IM_) of 10 um and a length (*L*_IM_) of 50 µm. The area ratio between the MOS transistor and MFM capacitor (A_MOS_:A_MFM_) is set to 10. b) Schematic diagram of the MFMIS FeFET with erase state (high *V*_th_) and program state (low *V*_th_), respectively. c) Conduction mechanism of the MFMIS FeFETs with erase and program states. d–g) Double‐sweep *I*_D_‐*V*_GS_ of the MFMIS FeFETs with different A_MOS_:A_MFM_ ratios (Sweep rate = 1 V/ms). The black and red symbols represent the forward and backward sweep measurements of the devices, respectively.

First, the electrical characteristics of the MFMIS FeFETs with different ILs are investigated. **Figure** **2a** shows the double‐sweep transfer characteristics (*I*_D_‐*V*_GS_) of the MFMIS FeFETs with ZrO_2_ and HfO_2_ ILs, denoted by green and blue symbols, respectively. In the MFMIS FeFETs, the channel width and length are defined by the width (*W*_IM_) and length (*L*_IM_) of the inner metal, respectively. The inner metal has a width (*W*_IM_) of 10 µm and a length (*L*_IM_) of 50 µm. The area ratio between the MOS transistor and MFM capacitor (A_MOS_:A_MFM_) is fixed at 10 when we investigated the effects of IL materials on tensile stress. Both devices exhibit anticlockwise hysteresis, which is attributed to the ferroelectric polarization of HZO. When a negative bias voltage is applied to the top metal, the ferroelectric HZO is polarized downward (into the Si channel layer), depleting the carriers in the Si channel and increasing the threshold voltage (*V*_th_) of the device. Conversely, when a positive bias voltage is applied to the top metal layer, HZO is polarized upward (into the top metal layer), thereby reducing the *V*_th_. Figure 2b shows the schematics of the MFMIS FeFET with erase state (high *V*_th_) and program state (low *V*_th_), respectively. It is noteworthy that polarization not only affects the channel resistance but also modulates the contact resistance at the source/drain‐channel interface.^[^ ^31^, ^32^ ^]^ The conduction mechanism governing carrier transport is schematically illustrated in Figure 2c. A detailed explanation of the conduction mechanism of the device will be further elucidated through the results of the LFN analysis. Figure S2a (Supporting Information) shows the retention characteristics of the device for up to 10^6^ s, indicating negligible *V*_th_ shift. Figure S2b (Supporting Information) shows the program/erase endurance characteristics of the device. Pulses of ±6 V and a duration of 50 µs are applied during the cycling endurance test. The *V*_th_ of the FeFET exhibits no changes in either program or erase states up to 10^5^ cycles of the cycling stress. Such robust cycling endurance performance is attributed to the structural advantages of MFMIS FeFETs, where a smaller electric field is applied to the FE/IL interface than the MFIS FeFETs.^[^ ^33^, ^34^ ^]^

The MFMIS FeFET with the HfO_2_ IL exhibits a much larger memory window (2.76 V) than the MFMIS with the ZrO_2_ IL (1.86 V). This disparity is attributed to the different strain levels applied to the ferroelectric layer (HZO) during the PMA process. In the PMA process, the difference in CTE between the films induces thermal strain (*ε*_th_), whose magnitude is expressed as^[^ ^35^ ^]^

(1) $$\varepsilon_{\mathrm{th}}=\int_{T_{0}}^{T}\left[\alpha_{s}-\alpha_{f}\right]\;dT$$

where *α*_s_ and *α*_f_ denote the CTE of the substrate and the ferroelectric film, respectively, *T*_0_ is the initial temperature at which the film and substrate are in a stress‐free state, and *T* is the current temperature. When *ε*_th_ is positive, it results in the film experiencing tensile stress, causing a concave‐upward bending. Therefore, a decrease in the CTE of the bottom electrode or substrate leads to the transformation of the in‐plane strain from compressive to tensile. Previous studies have reported that the application of tensile stress induces the formation of a polar o‐phase in HfO_2_, thereby improving its ferroelectric properties. It was noted that the CTE of HfO_2_ (8.5 × 10^−6^/°C) is significantly smaller than that of ZrO_2_ (12.5 × 10^−6^/°C).^[^ ^36^ ^]^ Consequently, in the MFMIS with the HfO_2_ IL, a larger tensile stress is exerted on the HZO layer during the PMA, leading to the enhancement of the anticlockwise memory window. The MFMIS FeFET with the HfO_2_ IL exhibits a much larger memory window (2.54 V) than the MFMIS with the ZrO_2_ IL (1.38 V). Note that the MW are extracted at *I*_D_ = 1nA. However, an intriguing observation is that there is a significant difference in the *I*_D_‐*V*_GS_ beyond just the memory window. Specifically, the MFMIS FeFET with HfO_2_ IL exhibits a substantially smaller on‐current (*I*_on_) and larger subthreshold swing (*SS*) than the device with ZrO_2_ IL. In MFMIS FeFETs with poly‐Si channels, both the FE layers and the poly‐Si channels are influenced by the strain applied during the PMA process. Whereas a higher magnitude strain can enhance remnant polarization, it can also cause damage to poly‐Si, resulting in significant degradation of the electrical properties (Supplementary Note 1). Hence, it is important to consider the tensile stress applied to poly‐Si when optimizing the strain in FETs because a higher magnitude strain is not always advantageous. This finding emphasizes the need to optimize the tensile strain during the PMA process instead of solely maximizing it.

#### 2.2.2 Effects of the Geometry on Strain

Next, we investigate the effect of geometrical factors on the strain in the MFMIS FeFETs. It should be noted that the MFMIS FeFETs with the ZrO_2_ IL are mainly utilized in the subsequent part of the study. This is due to the low *I*_on_ and unstable electrical properties exhibited by the MFMIS FeFETs with HfO_2_ IL, which hinder the measurement of LFN and accurate analysis. The A_MOS_:A_MFM_ ratio is modulated to examine the dependency on geometry on electrical characteristics. The voltage applied to the ferroelectric HZO layer is influenced by variations in the A_MOS_:A_MFM_ ratio, which directly affect the capacitance matching between the MOS capacitance (*C*_MOS_) and the ferroelectric capacitance (*C*_MFM_). The capacitance ratio can be expressed as:

(2) $$\frac{C_{MOS}}{C_{MFM}}=\frac{\varepsilon_{MOS}\frac{A_{MOS}}{d_{MOS}}}{\varepsilon_{MFM}\frac{A_{MFM}}{d_{MFM}}}$$

where *C* represents the capacitance, *ε* is the dielectric constant of the respective layer, *A* denotes the area, and *d* is the thickness of the dielectric. According to (1), the capacitance ratio (C_IG_/C_TG_) is proportional to the area ratio (A_MOS_/A_MFM_). Thus, the voltage applied to the ferroelectric layer increases with increasing A_MOS_/A_MFM_.

Figure 2d–g show the double‐sweep *I*_D_‐*V*_GS_ curves of the MFMIS FeFETs with different A_MOS_:A_MFM_ ratios. Note that power line cycle value of 3 is used during the measurement. The black and red symbols represent the forward and backward sweep measurements of the devices, respectively. The corresponding output characteristics (*I*_D_‐*V*_DS_) of the devices are shown in Figure S3a–d (Supporting Information). In the devices with A_MOS_:A_MFM_ ratio (*L*_IM_: *L*_TM_) = 10 (50 µm: 5 µm), 5 (50 µm: 10 µm), and 2 (50 µm: 25 µm), the FETs exhibit anticlockwise hysteresis behavior, where the memory window increases with an increase of the A_MOS_:A_MFM_. As the A_MOS_:A_MFM_ ratio is increased by a decrease of *L*_TM_, the *V*_GS_ coupled to the ferroelectric HZO layer is increased owing to the decreased capacitance of the MFM. This increase in the voltage coupled to MFM leads to the enlargement of the memory window in the MFMIS FeFETs, as shown in Figure 2d–f. With further decrement in the A_MOS_:A_MFM_, the device exhibits clockwise hysteresis, as shown in Figure 2g. When the A_MOS_:A_MFM_ becomes too small (A_MOS_:A_MFM_ (*L*_IM_: *L*_TM_)) = 1 (50 µm: 50 µm), the *V*_GS_ applied to the ferroelectric HZO layer is significantly decreased, resulting in a positive *V*_th_ shift in the program state. This shift is attributed to the trapping of the carriers into the defects inside the IL.

The increase in the ferroelectric memory window owing to the increase of A_MOS_:A_MFM_ ratio has been widely demonstrated in previous studies. However, in contrast to previous studies, there are notable differences in the fabricated MFMIS FeFETs with poly‐Si channels. In MFMIS FeFETs with single crystalline silicon (*c*‐Si), the degradation of *I*_on_ and SS is observed with an increase in the A_MOS_:A_MFM_ ratio.^[^ ^37^ ^]^ This is due to the increased effects of remote phonon scattering caused by the polarization of the HZO. Materials with high dielectric constant, such as FE HZO, consist of atomic groups that are easily polarized.^[^ ^38^ ^]^ As a consequence, channel electron scattering becomes more pronounced, leading to the degradation of channel mobility and subsequent decrease in *I*_on_. However, such degradation is not observed in the MFMIS FeFETs with a poly‐Si channel. Instead, a slight increase in *I*_on_ and a decrease in *SS* are observed. These behaviors cannot be explained solely by the different polarization states of the devices. It appears that there is a change in the properties of the poly‐Si material due to the geometrical factors of the MFMIS FeFETs during PMA. These findings highlight the necessity for further research to gain a comprehensive understanding of the underlying mechanisms and investigate the changes in greater detail.

### 2.3 LFN Analysis

#### 2.3.1 Bias‐Dependent LFN Characteristics of the FeFETs

To investigate the impact of geometrical factors on the MFMIS FeFETs, LFN analysis is conducted. The LFN measurement, also known as LFN spectroscopy, is a highly useful technique for studying strain effects in FeFETs. This technique offers exceptional sensitivity to defects in electronic devices, allowing detailed analysis of their electrical characteristics. Unlike conventional measurements, LFN spectroscopy can detect subtle changes in device performance that may not be easily observed using other methods.^[^ ^30^ ^]^ One of the key advantages of LFN spectroscopy is its non‐destructive nature, which enables the repeated monitoring of electrical characteristics under different conditions. This facilitates a comprehensive understanding of the device performance and the effects of strain. Moreover, LFN spectroscopy has been successfully utilized to analyze the characteristics of poly‐Si with various defects caused by different types of mechanical strains. Thus, by utilizing LFN spectroscopy to analyze MFMIS FeFETs, the geometrical effects on poly‐Si FETs can be interpreted.

**Figure 3:**
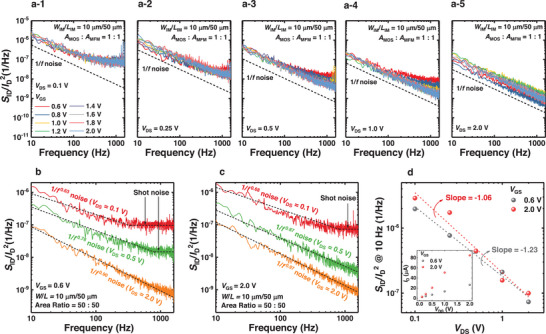
Drain current normalized PSD (*S*_ID_/*I*_D_^2^) versus frequency (*f*) of the FeFETs for various *V*_GS_ values measured at *V*_DS_ of a‐1) 0.1, a‐2) 0.25, a‐3) 0.5, a‐4) 1.0, and a‐5) 2.0 V, respectively. *S*_ID_/*I*_D_^2^ versus *f* of the FeFET measured at different *V*_DS_ values for *V*_GS_ values of b) 0.6 and c) 2.0 V, respectively. d) *S*_ID_/*I*_D_^2^ sampled at 10 Hz versus *V*_DS_ for different values of *V*_GS_. The inset shows the *I*_D_ versus *V*_DS_ in each *V*_GS_ condition.

**Figure 4:**
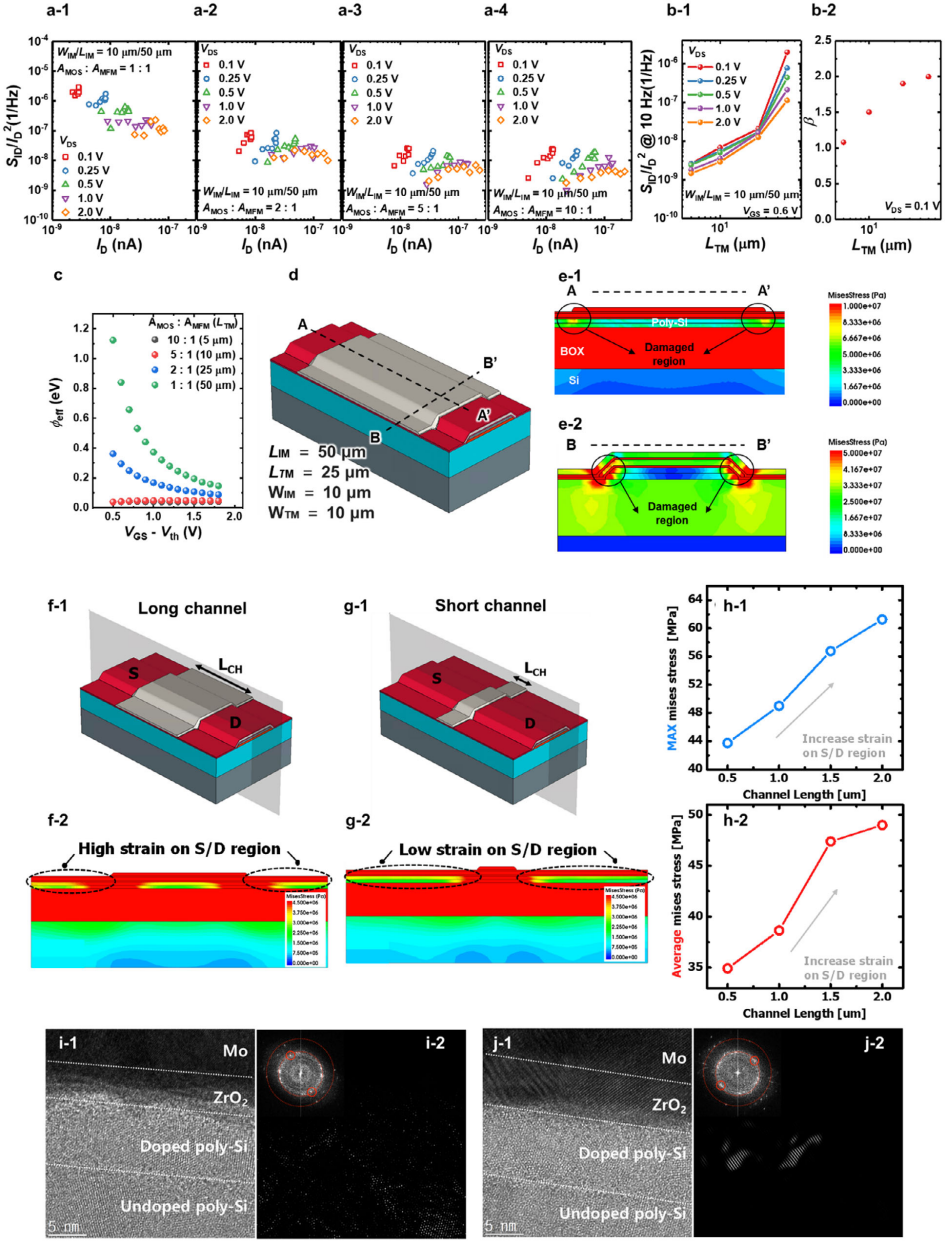
*S*_ID_/*I*_D_^2^ sampled at 10 Hz versus *I*_D_ measured at different *V*_GS_ values of the FeFETs for A_MOS_: A_MFM_ = a‐1) 1, a‐2) 2, a‐3) 5, and a‐4) 10, respectively. In all cases, the MFMIS FeFETs exhibit a strong *V*_DS_ and *V*_GS_ dependencies. b‐1) *S*_ID_/*I*_D_^2^ sampled at 10 Hz verus *L*_TM_ with *V*_DS_ a parameter. b‐2) *β* versus LTM of the MFMIS FeFETs. Note that *β* is extracted at *V*_DS_ = 0.1 V, where the contact resistance is dominant. c) Relationship between the *Φ*_eff_ and *V*_GS_ for the FeFETs with varying A_MOS_:A_MFM_. Notably, an increase in *Φ*_eff_ is observed as *L*_TM_ increases (corresponding to a decrease in A_MOS_:A_MFM_). d) 3D bird's‐eye view of the device used in the TCAD simulation. The magnitude of the Mises stress applied to the device along the cross‐section of the device e‐1) A‐A' direction and e‐2) B‐B’ direction in Figure 4(d)). 3D bird's‐eye view of the poly‐Si FeFETs with MFMIS structure having channel length (*L*_ch_) of f‐1) 2 µm and f‐2) 0.5 µm, respectively. Mises stress contour along the cross‐section of the poly‐Si FeFETs with MFMIS structure having *L*_ch_ of g‐1) 2 µm and g‐2) 0.5 µm, respectively. h‐1) Max mises stress and h‐2) average Mises stress, respectively, as a function of channel length. Cross‐sectional HR TEM images of FeFETs with long and short *L*_TM_ are shown in Figures 4(i‐1) and (j‐1), respectively. Note that the HR TEM measurement is conducted at the edge region of the device, as indicated by the LFN analysis and TCAD simulation, which demonstrated that the damage is concentrated in the edge region of the device. Inverse FFT images constructed using the diffraction peak (indicated by red circles) are shown in Figures 4(i‐2) and (j‐2), with insets displaying the FFT of the corresponding area.

**Figure 5:**
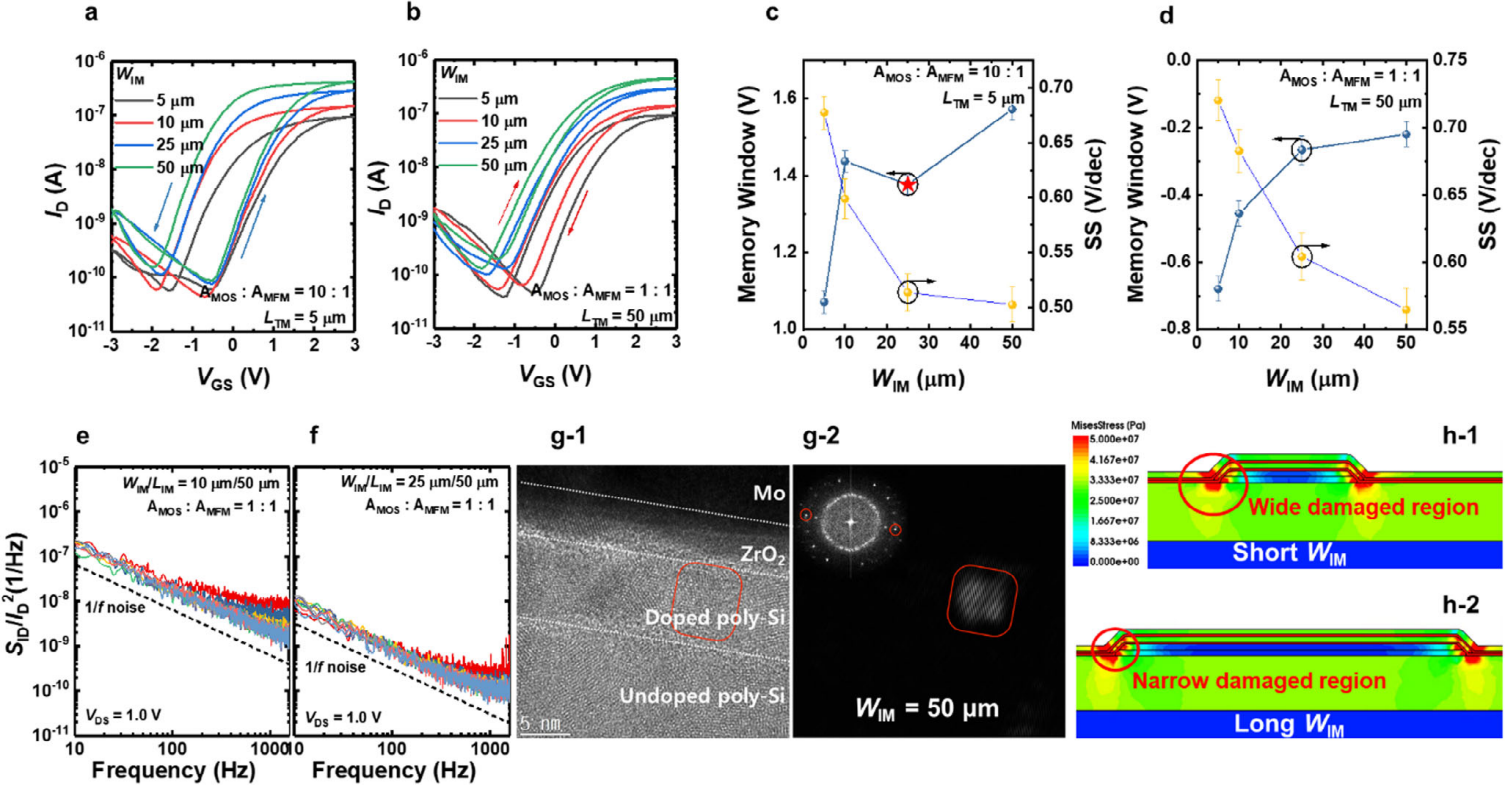
a) *I*_D_‐*V*_GS_ characteristics of FeFETs with *L*_TM_ of 5 µm and various *W*_IM_ values. All devices exhibit anticlockwise hysteresis, which originates from the polarization of the ferroelectric material. b) *I*_D_‐*V*_GS_ characteristics of FeFETs with *L*_TM_ of 50 µm and various *W*_IM_ values. These devices exhibit clockwise hysteresis, the magnitude of which increases as *W*_IM_ decreases. Key electrical parameters, including the memory window and SS, for devices with *L*_TM_ of c) 5 µm and d) 50 µm, with varying *W*_IM_. Note that the five different MFMIS FeFETs are measured. *S*_ID_/*I*_D_^2^ versus frequency of the device with *W*_IM_ of e) 10 and f) 25 µm, respectively. The *V*_DS_ is set to 1.0 V, and the *V*_GS_ is varied to observe the change of *S*_ID_/*I*_D_^2^ with *I*_D_. g‐1) HR TEM image and g‐2) Inverse FFT image of the FeFETs with larger *W*_IM_, respectively. The magnitude of Mises strain of the device with h‐1) short and h‐2) long *W*_IM_, respectively.

The source of LFN spectroscopy should be clearly identified when using LFN spectroscopy for the analysis of the geometric effects on FeFETs. The most accurate way to determine the source of LFN in FETs is to investigate the bias dependency of the power spectral density (PSD) in the devices. In this study, the PSDs are measured at different values of *V*_GS_ and *V*_DS_ to determine the origin of LFN in MFMIS FeFETs. **Figure** **3**a‐1–a‐5 show the drain current normalized PSD (*S*_ID_/*I*_D_^2^) versus frequency (*f*) of the FeFETs for various *V*_GS_ for *V*_DS_ values of 0.1, 0.25, 0.5, 1.0, and 2.0 V, respectively. Note that the device with *W*_IM_/*L*_IM_ = 10 µm/50 µm and *W*_TM_/*L*_TM_ = 10 µm/50 µm is used in this experiment. In all the bias conditions, the FeFETs exhibit 1/*f* ^γ^ noise behavior with *γ* = − ln*S*_ID_/ln*f*. Figure 3b shows the *S*_ID_/*I*_D_^2^ versus *f* of the FeFET measured at different *V*_DS_ values for a fixed *V*_GS_ of 0.6 V. *S*_ID_/*I*_D_^2^ of the device exhibits very high sensitivity to *V*_DS_. At lower *V*_DS_ values, the shot noise is observed in the high *f* ranges (*V*_DS_ = 0.1 V, *f* > 500 Hz and *f* > 800 Hz for *V*_DS_ = 0.5 V, and full 1/*f* noise for *V*_DS_ = 2.0 V). With an increase in *V*_DS_, *S*_ID_/*I*_D_^2^ decreases significantly, and the 1/*f* noise plays a more dominant role. Figure 3c shows the *S*_ID_/*I*_D_^2^ of the device measured at different *V*_DS_ values for a fixed value of *V*_GS_ at 2.0 V. In this case, an overall increase in 1/*f* noise is observed with an increase in *V*_GS_. Figure 3d shows the *S*_ID_/*I*_D_^2^ sampled at 10 Hz versus *V*_DS_ as the parameter of *V*_GS_. The inset shows the *I*_D_ versus *V*_DS_ in each value of *V*_GS_.

The carrier number fluctuation (CNF) model is commonly used to explain the 1/*f* noise behavior in FETs, including FeFETs.^[^ ^39^, ^40^ ^]^ In this model, 1/*f* noise arises from the random trapping and detrapping of carriers to and from defects present within the gate oxide. It has been observed that the captured carriers induce additional Coulombic scattering, leading to correlated mobility fluctuations (CMF) in the channel carriers.^[^ ^41^, ^42^ ^]^ The CNF model, extended to include CMF, can be expressed as follows:^[^ ^41^ ^]^

(3) $$\frac{S_{ID}}{{I_{D}}^{2}}={\left(\frac{g_{m}}{I_{D}}\right)}^{2}\;S_{Vfb}{\left(1\pm \alpha_{C}\mu_{eff}C_{OX}\frac{I_{D}}{g_{m}}\right)}^{2}$$

where

(4) $$S_{Vfb}=\frac{q^{2}kT\lambda N_{T}}{fWL{C_{OX}}^{2}}$$

where *g*_m_ is the transconductance, *S*_Vfb_ is the flat band voltage fluctuation, α_*C*_ is the Coulombic scattering parameter in the CMF model, µ_*eff*_ is the effective mobility, *C*_OX_ is the oxide capacitance per unit area, *q* is the electron charge, *T* is the absolute temperature, λ is the oxide tunneling distance, *N*_T_ is the volume trap density, and *W* and *L* represent the width and the length of the FET channel. According to the CNF model, *S*_ID_/*I*_D_^2^ should decrease with an increase in *I*_D_ following the behavior of *g*_m_/*I*_D_ whose value is inversely proportional to SS. In particular, in the linear region, the decrease in *S*_ID_/*I*_D_^2^ is significant. However, the FeFET with MFMIS structure does not exhibit such characteristics. Instead, *S*_ID_/*I*_D_^2^ exhibits an increase with respect to the increase in *I*_D_ in the low *V*_DS_ region and remains the same value at the high *V*_DS_ values. This behavior cannot be explained by the CNF with CMF model. Moreover, the PSD of the device exhibits a very high sensitivity to *V*_DS_. With an increase in *V*_DS_, *S*_ID_/*I*_D_^2^ decreases exponentially, as shown in Figure 3d. This behavior also cannot be accounted for by CNF, which does not exhibit significant *V*_DS_ dependency.

In FETs with poly‐Si channels, the Schottky barrier (SB) contact at the source/drain‐channel junction plays a crucial role in noise generation. Barrier height fluctuation (BHF) is a phenomenon observed at metal‐semiconductor junctions, where the energy barrier controlling electron injection experiences random variations. These fluctuations primarily arise from the random distribution of defects at the interface between the metal and the semiconductor, which introduce local electrostatic potential fluctuations. Such variations affect the effective Schottky barrier height (SBH), modulating carrier injection and increasing LFN. In FeFETs, the source/drain SBH plays a crucial role in determining carrier transport characteristics, making it highly susceptible to external factors such as strain effects, charge trapping, and interface defects. Since BHF directly affects electron flow across the junction, it contributes significantly to 1/*f* noise in FeFETs. Therefore, any modification to the geometrical parameters that alter strain distribution or charge trapping at the interface will influence BHF and, consequently, noise levels. The BHF in the SB contact is represented as:^[^ ^44^ ^]^

(5) $$\frac{S_{ID}}{{I_{D}}^{2}}=A\eta^{2}{\left(\frac{q}{kT}\right)}^{2}\frac{1}{{\left(1-e^{-q\eta \frac{V_{DS}}{kT}}\right)}^{2}}\frac{1}{f}$$

where A (in square volts) is a parameter for noise amplitude proportional to defect density at the SB contact, and η = *R*_SB_/(*R*_SB_+*R*_C_), where *R_SB_* and *R*_C_ are the SB and channel resistance, respectively. As shown in Equation (4), the *S*_ID_/*I*_D_^2^ exhibits an exponential relationship with *V*_DS_, which explains the 1/*f* noise behavior of the FeFET. The LFN spectroscopy confirms that the conduction of the FeFET is determined by the SB and not by the channel in the measured *I*_D_ range.

#### 2.3.2 Effects of *L*_TM_ and *W*_IM_ on LFN Characteristics of the FeFETs

Based on these results, the LFN characteristics of FeFETs are compared for different A_MOS_:A_MFM_ ratios. First, we change the *L*_TM_ to control the A_MOS_:A_MFM_ ratio while maintaining *L*_IM_ at 50 µm. Note that the *W*_IM_ and the width of the top metal (*W*_TM_) are fixed at 10 µm when investigating the effects of *L*_TM_ on the performance of MFMIS FeFETs. **Figure** **4**a‐1–a‐4 show *S*_ID_/*I*_D_^2^ sampled at 10 Hz versus *I*_D_ measured at different *V*_GS_ values of the FeFETs with A_MOS_:A_MFM_ = 1, 2, 5, and 10, respectively. In all cases, the MFMIS FeFETs exhibit strong *V*_DS_ and *V*_GS_ dependencies. The origin of these dependencies is explained in Note S2 and Figure S5 (Supporting Information). Figure 4b‐1 shows *S*_ID_/*I*_D_^2^ sampled at 10 Hz versus *L*_TM_ for different parameter values of *V*_DS_. Interestingly, the PSD of the FeFETs significantly decreases for increasing A_MOS_:A_MFM_ ratios (decrease in *L*_TM_). Although the sensitivity of 1/*f* noise to changes in the size of the conductive channel has been widely recognized in previous studies,^[^ ^41^, ^42^, ^43^, ^44^ ^]^ the devices examined in this study exhibit distinct noise behaviors primarily driven by Schottky barrier (SB) fluctuations, even when the conductive channel size remains constant, depending on the length of beam metal. This highlights the influence of geometrically varying strain effects on SB‐dominated noise. A recent study reported that the LFN characteristics of the MFMIS FEFETs with IGZO channel vary depending on A_MOS_:A_MFM_ ratios;^[^ ^45^ ^]^ however, the 1/*f* noise increases with an increase in the A_MOS_:A_MFM_, which is attributed to the increase in remote phonon scattering due to the increased polarization. However, the FeFETs with poly‐Si channels in this study exhibit contrary results. This suggests that another factor, such as strain stress, plays a crucial role. The channel width and length of the FeFETs do not change with the A_MOS_:A_MFM_. Thus, it can be assumed that the change in the SB contact in the FeFET stems from the change in strain effects due to the change in *L*_TM_. Figure 4b‐2 shows the slope of the plot shown in Figure 4b‐1, which is defined as *β* = ∂ln(*S*_ID_/*I*_D_^2^)/∂ln(*I*_D_). Note that *β* is extracted at *V*_DS_ = 0.1 V. The FeFETs with longer *L*_TM_ exhibit larger values of *β*. This is because the transition of the noise source from the channel bulk to the contact resistance is more pronounced in devices with shorter *L*_TM_, which have smaller contact resistance due to a lower effective Schottky barrier height (SBH). In contrast, devices with longer *L*_TM_ exhibit larger contact noise due to a higher effective SBH, with 1/*f* noise predominantly determined by contact noise across most operating regions. As a result, the change in *S*_ID_/*I*_D_^2^ relative to *I*_D_ becomes negligible, leading to a smaller *β* value. It is widely known that the strain effects are dependent on the CTE of materials, as shown in Figure 2a. However, even when employing the same gate stack materials in FeFETs, the strain effects can show dependence on the geometry of the gate stack. Specifically, *L*_TM_ plays a crucial role in the strain effects observed in FETs. In cases where the *L*_TM_ is short, the strain effect is usually insignificant because the strain induced by the metal remains localized in a small area.^[^ ^46^ ^]^ However, as the length of the *L*_TM_ increases, the strain effect becomes more pronounced as the mechanical strain spreads over a larger area. However, in addition to the FE layer, a poly‐Si layer should be considered.

Previous studies have reported that strain significantly affects poly‐Si and its properties, such as grain size, defects at grain boundaries, and surface protrusions.^[^ ^47^ ^]^ For a longer *L*_TM_, a greater strain is exerted on the edge of the top metal. Consequently, the SB region in a FeFET with a longer *L*_TM_ may be subject to increased strain effects, potentially resulting in damage and an associated increase in 1/*f* noise. An increased density of defects in the SB contact can lead to an increase in the SB height. The SB height can be determined by analyzing the Arrhenius plot of ln(*I*_D_/*T* ^2^) versus 1000/*T*, as depicted in Figure S6a–d (Supporting Information). Note that the intrinsic SBH is generally considered temperature‐independent over a narrow range like 20–80 °C. However, effective SBH (*Φ*_eff_) extracted from temperature‐dependent current‐voltage (*I*–*V*) measurements may appear to decrease with increasing temperature due to effects such as thermionic emission and barrier lowering. Therefore, temperature‐dependent measurements were used to account for these effects when extracting the *Φ*_eff_. Figure 4c shows the relationship between the *Φ*_eff_ and *V*_GS_‐*V*_TH_ for the FeFETs with varying A_MOS_:A_MFM_. The *Φ*_eff_ extraction is performed in the high *V*_TH_ state. Devices with larger *L*_TM_ exhibit a greater shift in Vth due to the more dominant charge trapping. Notably, an increase in *Φ*_eff_ is observed as *L*_TM_ increases (corresponding to a decrease in the A_MOS_:A_MFM_). Moreover, for the FeFETs with longer *L*_TM_ (*L*_TM_ = 25 and 50 µm), the *Φ*_eff_ exhibits a strong dependence on *V*_GS_, while the FeFETs with shorter *L*_TM_ (*L*_TM_ = 5 and 10 µm) exhibit almost no dependence. This is because in the case of longer *L*_TM_, the contact resistance is large, and thus, most of the voltage drop is applied to the contact region, and thus the *Φ*_eff_ decreases with increasing *V*_GS_. Contrarily, as the contact resistance is relatively smaller than the bulk channel resistance in the shorter *L*_TM_, most of the voltage is applied to the bulk region, thereby resulting in the negligible dependence of *Φ*_eff_ on *V*_GS_. The variation in the relationship between *Φ*_eff_ and *V*_GS_ depending on the *A*_MOS_: *A*_MFM_ ratio is also a phenomenon that cannot be explained solely by differences in capacitance due to the area ratio. This highlights the necessity of considering the effects of geometry on strain when analyzing the operation of MFMIS FeFETs. The LFN characteristics of the devices are also measured at various temperatures. Figure S7 (Supporting Information) illustrates the S_ID_/*I*_D_^2^ PSD of the devices measured at different *V*_DS_ values and temperatures. As the temperature increases, resulting in a decrease in SB height, the 1/*f* noise decreases. Figure S8 (Supporting Information) shows the *S*_ID_/*I*_D_^2^ sampled at 10 Hz as a function of *V*_GS_, exhibiting temperature dependence. The open and solid symbols correspond to the cases of *V*_DS_ of 0.1 and 1.0 V, respectively.

To further assess the effect of the device geometry on strain in the FeFETs, a TCAD simulation is carried out. Figure 4d illustrates a 3D bird's‐eye view of the device used in the TCAD simulation. Figure 4e‐1 shows the magnitude of the von Mises stress applied to the device along the cross‐section of the device (A‐A' direction in Figure 4d), indicating that the strain is concentrated at the edge region where the top metal is deposited. Consequently, with a longer *L*_TM_, the strain exerted on the source and drain regions of the device is intensified, leading to increased SB height and 1/*f* noise. Additionally, Figure 4e‐2 displays the magnitude of the mises stress along the width direction (B‐B' direction in Figure 4d), showing that the strain is also concentrated at the edge region. The mapping of strain obtained from the TCAD simulation is in alignment with the LFN measurement results, emphasizing the importance of considering the geometric factor in FeFETs. To further support our analysis, we conducted strain simulations at smaller device dimensions. Figure 4f‐1,f‐2 show 3D bird's‐eye view of the poly‐Si FeFETs with MFMIS structure having channel length (*L*_ch_) of 2 µm and 0.5 µm, respectively. Figure 4g‐1,g‐2 show mises stress contour along the cross‐section of the poly‐Si FeFETs with MFMIS structure having *L*_ch_ of 2 and 0.5 µm, respectively. It is clearly observed that the longehr *L*_ch_ induces more strain on the source/drain region. Figure 4h‐1,h‐2 show the strain simulation results for MFMIS FeFETs as a function of *L*_ch_ with downscaled devices. The results demonstrate that both maximum and average Mises stress increase as the channel length decreases, confirming that geometrical factors significantly influence strain‐induced effects.

Cross‐sectional high‐resolution transmission electron microscopy (HR TEM) images of FeFETs with long and short *L*_TM_ are shown in Figure 4i‐1,j‐1, respectively. Note that the HR TEM measurement is made at the edge region of the device, as indicated by the LFN analysis and TCAD simulation, which demonstrated that the damage is concentrated at its edge region (Figure S9, Supporting Information). HR TEM images show that strain stress not only affects the electrical characteristics of the poly‐Si channel but also causes physical damage to the device edge region. The inverse fast Fourier transform (FFT) images constructed using the diffraction peak (indicated by red circles) are shown in Figure 4i‐2,j‐2, with insets displaying the FFT of the corresponding area. The device with the long *L*_TM_ exhibits a lower intensity of the diffraction peaks marked by red circles, indicating grain deformation and lower quality. In contrast, the device with short *L*_TM_ displays larger and better grain crystallinity, indicating a significant reduction in strain‐induced damage during the PMA process. To extend our discussion to downscaled devices, strain stress analysis is conducted through TCAD simulations on devices with smaller channel sizes (Method).

In addition to *L*_TM_, the width of the metals, including *W*_TM_ and *W*_IM_, which have the same magnitude in the devices, also plays a crucial role in determining the effects of geometrical strain. Therefore, we also investigate the effects of width variation on the electrical and strain effects on the MFMIS FeFETs. Here, we change the *W*_IM_ value. **Figure** **5**a shows the *I*_D_‐*V*_GS_ characteristics of FeFETs for *L*_TM_ of 5 µm and for various *W*_IM_ values. It is worth noting that all devices exhibit anticlockwise hysteresis, which originates from the polarization of the ferroelectric material. With an increase in *W*_IM_, a significant improvement in electrical properties is observed. Similar trends are observed in devices with longer *L*_TM_ (50 µm), as shown in Figure 5b. It is important to note that devices with longer *L*_TM_ exhibit clockwise hysteresis, and its magnitude increases as *W*_IM_ decreases. The key electrical parameters, including the memory window and SS, for devices with *L*_TM_ of 5 and 50 µm, with varying *W*_IM_, are summarized in Figure 5c,d, respectively. In both cases, devices with larger *W*_IM_ demonstrate significantly better electrical characteristics. The slight decrease in the memory window observed in Figure 5c (marked by a red star) is attributed to the steep decrease in SS. Based on these results, it can be inferred that increasing *W*_IM_, in contrast to *L*_TM_, reduces the damage caused by strain.

Figure 5e,f show the plots of *S*_ID_/*I*_D_^2^ versus frequency of the device with *W*_IM_ of 10 and 25 µm, respectively. Here, *V*_DS_ is fixed at 1.0 V and *V*_GS_ is varied to observed the change in *S*_ID_/*I*_D_^2^ with *I*_D_. A significant decrease in the 1/*f* noise is observed with an increase in *W*_IM_, further demonstrating an improvement in the SB characteristics. Figure 5(g‐1) and (g‐2) show the HR TEM image and inverse FFT image of the FeFETs with a larger *W*_IM_. Contrary to the devices with shorter *W*_IM_ (Figure 4(g‐2) and (h‐2)), clear and highly crystalline grains are observed, demonstrating decreased damage to the devices. Figure 5(h‐1) and (h‐2) show the magnitude of von Mises strains of the devices with short and long *W*_IM_, respectively. Strain is applied in the narrower region at the edge of the device with a longer *W*_IM_. It appears that the tensile stress is mitigated by the increase in *W*_IM_.

In summary, *L*_TM_ and *W*_IM_ play crucial roles in determining strain effects and Schottky barrier fluctuations in MFMIS FeFETs. A longer *L*_TM_ increases strain at the source/drain junctions, leading to a higher density of localized defects, which in turn exacerbates Schottky barrier height fluctuations and increases LFN. This results in the reverse scaling effect, where the noise decreases with a decrease in the geometric length. Conversely, a wider *W*_IM_ helps distribute mechanical stress more evenly, reducing localized strain effects and lowering noise levels. In contrast to previous studies that primarily focused on the materials aspects of strain engineering, this study emphasizes the significance of geometrical effects on the strain in FeFETs. The length and width of the metal beam within the device directly influence the applied strain, leading to variations in electrical properties. Therefore, it is crucial to consider the geometrical factors during strain engineering in next‐generation ferroelectric‐based devices, such as 3D VNAND FeFETs.

## 3 Conclusion

We investigated the geometrically varying strain effects and their influence on the device performance of poly‐Si FeFET with MFMIS structure via LFN spectroscopy. By varying the length and width of the gate metal beams, we applied different levels of strain and observed longer *L*_TM_ values increased strain stress in the top metal, resulting in a reverse scaling effect where noise increased with the channel length, contrary to conventional FET behavior. While greater strain can enhance ferroelectricity, it also causes degradation in the poly‐Si channel, negatively affecting device performance. Our results show that optimizing geometrical parameters, particularly using shorter *L*_TM_ and wider *W*_IM_, can mitigate strain‐induced damage and improve overall device performance. This work underscores the importance of balancing strain effects through careful geometrical and material design in FeFETs, providing valuable guidelines for enhancing performance and reliability in future applications.

## 4 Experimental Section

### Fabrication Process of FeFETs

The JL TFTs were fabricated on a thermally grown 400nm‐thick SiO_2_ BOX layer on Si substrate. First, a 30 nm‐thick undoped poly‐Si layer and then a 10 nm‐thick phosphorous (P) doped poly‐Si layer were deposited using low‐pressure chemical vapor deposition at 620 °C, respectively. The multilayer poly‐Si structure was adopted for full channel depletion of poly‐Si without degradation of ferroelectricity of HZO. Afterward, rapid thermal annealing (RTA) was performed at 900 °C for 30 s under N2 ambient conditions to activate the dopant on doped poly‐Si. Followed by standard cleaing‐1 (SC‐1), ZrO_2_/Mo/HZO/Mo was deposited sequentially for the top‐gate, ferroelectric, inner‐gate, insulator, and semiconductor (MFMIS) gate stacks. For higher ferroelectricity of HZO without degradation of the IL, the inner and top metals were patterned in different areas. ZrO_2_ and HZO were formed by atomic layer deposition as an interlayer dielectric (IL) and ferroelectric (FE), respectively. 80 cycles of ZrO_2_ were deposited using tetrakis (ethylmethylamino) zirconium (TEMA‐Zr) as the Zr precursor and O_3_ as the oxidant. Next, a 30 nm‐thick Mo was sputtered as the inner‐gate metal. Above the inner gate, HZO was deposited using TEMA‐Zr, TEMA‐Hf, and O_3_. The HZO film was deposited by alternating cycles of TEMA‐Zr and TEMA‐Hf in a ratio of 2:1, corresponding to an overall Hf:Zr ratio of 2:1. This specific composition was chosen because pure HfO₂ typically exhibits paraelectric behavior, while pure ZrO₂ shows antiferroelectric characteristics.^[^ ^48^ ^]^ At the Hf:Zr ratio of 2:1, the HZO film demonstrates robust ferroelectric behavior, which was essential for the device operation. The cycle was repeated 26 times to target an 8nm‐thick HZO layer, after which a 50 nm‐thick Mo was sputtered as the top‐gate metal. Subsequently, a PMA was performed at 500 °C for 30 s under N_2_ ambient conditions using RTA to reveal the orthorhombic phase (O‐phase) of HZO. Then, a 300 nm‐SiO_2_ was deposited for a passivation layer using plasma‐enhanced vapor chemical deposition. Finally, the metal contacts and pads were deposited with Mo using the sputter after etching the contact holes using reactive ion etching. Phosphorus ions were implanted on the source/drain region with a dose of 10^15^ cm^−2^ and energy of 10 keV (Figure S1f, Supporting Information). PMA was performed using RTA at 500 °C for 30 s in N_2_ ambient to crystallize HZO film and activate dopants. Finally, high‐pressure annealing (HPA) was conducted to improve the ferroelectricity of FeFETs. HPA was maintained at 400 °C in the forming gas ambient conditions (H_2_: 4% and N_2_: 96%) for 30 min.

### Electrical Measurement

The ferroelectricity of the FeFETs using a parameter analyzer (Keithley 4200‐SCS) and current‐voltage module (4225‐PMU) was investigated. The *P*–*V* curves were measured using the PUND method in conjunction with a time‐transient measurement using a triangular pulse with a frequency of 2.5 kHz.

### LFN Measurement

To investigate the LFN characteristics of the metal‐ferroelectric‐metal‐insulator‐semiconductor MFMIS FeFETs, we employed a precise measurement setup incorporating a semiconductor parameter analyzer (B1500A), a low‐noise current amplifier (SR570), and a signal analyzer (35670A). The measurement methodology was designed to capture the drain current fluctuations with high sensitivity and accurately extract the power spectral density (PSD) through Fast Fourier Transform (FFT) analysis. In this setup, the gate bias was applied using the B1500A, ensuring precise control over the transistor's operating point. Simultaneously, the drain bias was applied through the SR570, which not only supplies the required voltage but also functions as a low‐noise current amplifier to enhance the signal‐to‐noise ratio.^[^ ^49^, ^50^ ^]^ The amplified drain current signal was then fed into the 35670A signal analyzer, which processes the time‐domain fluctuations and extracts the frequency‐dependent noise characteristics using FFT‐based PSD calculations. The selection of this measurement technique was based on the fundamental principle that, in FETs, the primary source of signal fluctuations originates from variations in the drain current.^[^ ^51^, ^52^ ^]^ Consequently, monitoring the drain current noise spectrum provides valuable insight into the underlying noise mechanisms, including carrier trapping/detrapping processes, Schottky barrier fluctuations, and bulk semiconductor noise contributions.

In this study, the frequency range for LFN measurements was carefully chosen to cover the spectrum where 1/*f* noise was most pronounced. Specifically, the measurement frequency window was set between 10 and 1600 Hz, ensuring that the noise behavior and its dependence on device parameters could be effectively analyzed. Furthermore, since Schottky barrier fluctuations were known to be highly sensitive to temperature variations, it was conducted noise measurements at multiple temperatures ranging from 20 to 80 °C. This allowed us to not only assess the temperature‐dependent variation in electrical characteristics but also to evaluate the impact of thermal fluctuations on the Schottky barrier height and its associated noise contributions.

### TCAD Simulation

A TCAD simulation was used to investigate the geometrically varying strain effects in MFMIS FeFETs. The Sentaurus interconnect tool was employed to map the magnitude of the von Mises stress to evaluate the overall stress state in a material with the same process sequence (Figure S1, Supporting Information) as the MFMIS FEFET, including PMA. In addition, the stress parameters of materials were rigorously set. 1) In molybdenum, the CTE was set to 5.5 × 10^−6^ /°C. The shear modulus, which was the material's resistance to deformation under shear stress, was defined as 13.4×10^10^ Pa. Furthermore, bulk modulus, which was the material's resistance to compression, is set to 28×10^10^ Pa. 2) In IL (HfO_2_), CTE was set to 8.5 × 10^−6^/°C. 3) In IL (ZrO_2_) CTE was applied to 12.5 × 10 ^−6^/°C.

## Conflict of Interest

The authors declare no conflict of interest.

## Supporting information



Supporting Information

## Data Availability

The data that support the findings of this study are available from the corresponding author upon reasonable request.
