# Supplementary material for: Low‐Frequency Noise Spectroscopy for Navigating Geometrically Varying Strain Effects in HfO2 Ferroelectric FETs
Source: Adv Sci (Weinh). 2025 Apr 2;12(23):2501367. doi: 10.1002/advs.202501367 (PMC12199404; doi:10.1002/advs.202501367)
Supplement: Supplementary file 1 — Supporting Information [file ADVS-12-2501367-s001.docx]

Supporting Information

**Low-Frequency Noise Spectroscopy for Navigating Geometrically Varying Strain Effects in HfO_2_ Ferroelectric FETs**

*Ryun-Han Koo^1^, Wonjun Shin^2,3^*, Sangwoo Kim^4^, Jangsaeng Kim^2^, Been Kwak^4^,* *Jiseong Im^1^, Hyunwoo Kim^5^, Deok-Hwang Kwon^6^, Suraj S. Cheema^3^, Jong-Ho Lee^1^, and Daewoong Kwon^4^**

^1^Department of Electrical and Computer Engineering and Inter-university Semiconductor Research Center, Seoul National University, Seoul 08826, Republic of Korea

^2^Department of Semiconductor Convergence Engineering, Sungkyunkwan University

^3^Research Laboratory of Electronics, Massachusetts Institute of Technology, Cambridge, MA, USA

^4^Department of Electrical Engineering, Hanyang University, Seoul 04763, Republic of Korea

^5^Department of Electrical and Electronics Engineering, Konkuk University, Seoul 27478, Republic of Korea

^6^Center for Energy Materials Research, Korea Institute of Science and Technology, Seoul 02792, Republic of Korea

R. H. Koo, W. Shin, S. Kim, and J. Kim contributed equally to this work.

* E-mail: swj0107@skku.edu, dw79kwon@hanyang.ac.kr

Keywords: HfO_2_ ferroelectric thin films; strain; low

Figure S1. Fabrication process of MFMIS FeFETs. (a) 30nm-thick undoped poly-Si and 10nm-thick P doped poly-Si were deposited and patterned as a active channel respectively on SiO_2_ BOX, followed by dopant activation using RTA. (b) ZrO_2_ was deposited as an interlayer dielectric using ALD. (c) 30nm-thick Mo was deposited and patterned as an inner-gate metal. (d) HZO was deposited as a ferroelectric layer using ALD. (e) 50nm-thick Mo was deposited and patterned as a top-gate metal, followed by a post metal annealing using RTA. (f) Metal contacts and pads were deposited and patterned with Mo after SiO_2_ passivation.

Figure S2. (a) Retention characteristics of the device upto 10^6^ s. Th *V*_th_ shift is negligible. (b) Program/erase endurance characteristics of the device. The $\pm$ 6 V of 50 μs pulses are applied during the cycling endurance test. The *V*_th_ of the FeFET exhibit no changes in both program/erase states upto 10^5^ times of cycling stress. Such robust cycling endurance performance is attributed to the structural advantages of MFMIS FeFETs where a smaller electric field is applied to the FE/IL interface compared to the MFIS FeFETs

Figure S3. Output characteristics of the MFMIS FeFETs with A_MOS_:A_MFM_ = (a) 1, (b) 2, (c) 5, and (d) 10.

Figure S4. Transfer characteristics of the MFMIS FeFETs with HfOx dielectric. The curves are measured at different AMOS : AMFM ratios.

Figure S5. *S*_I_, normalized by (black) *I*^2^ and (red) *g_m_^2^*, is plotted as a function of *V*_g_. *V*_d_ = 1 mV. The corresponding *I*–*V*_g_ curve is shown in the inset. The dashed line indicates the threshold voltage. This Figure is reproduced from [44] with the permission of IEEE Transactions on Electronic Devices.

Figure S6. Arrhenius plot of ln(*I*_D_/*T*^2^) versus 1000/*T* of the devices with A_MOS_:A_MFM_ = (a) 10:1, (b) 5:1, (c) 2:1, and (d) 1:1. The devices with smaller A_MOS_:A_MFM_ exhibit the stroner temperature dependce. From this relationship, the SB height can be extracted.

Figure S7. S_ID_/*I*_D_^2^ PSD of the devices measured at different *V*_DS_ values and temperatures. It is observed that as the temperature increases, resulting in a decrease in SB height, the 1/*f* noise decreases.

Figure S8. *S*_ID_/*I*_D_^2^ sampled at 10 Hz as a function of *V*_GS_, representing the temperature dependence. The open and solid symbols correspond to the cases of *V*_DS_ of 0.1 and 1.0 V, respectively.

Figure S9. TEM image of the MFMIS FeFET where the grain analysis is performed.


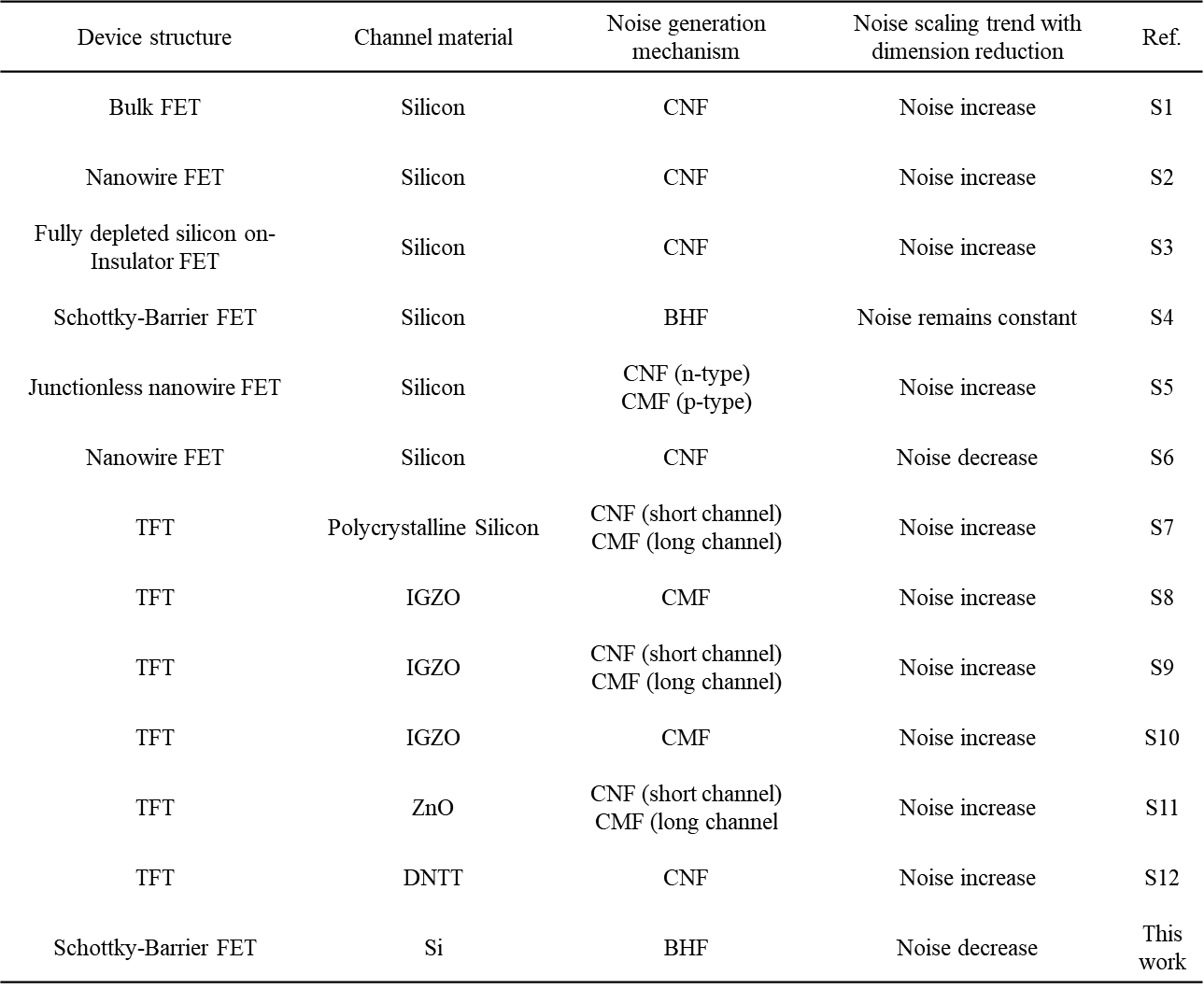


Table S1. Previous study on the channel dimension dependence of the LFN.

**Supplementary Note 1: Effects of IL on LFN characteristics of SB FETs**

Figure SN1(a) shows the *S*_ID_/*I*_D_² versus frequency of the FeFETs with different ILs with *V*_DS_ fixed at 0.1 V. Figure SN1(b) shows *S*_ID_/*I*_D_² versus *V*_GS_ for FeFETs with different ILs. Despite having the same channel material (poly-Si) and the only difference being the IL, the channel noise differs by more than three times between the two devices. This indicates that the poly-Si channel in the ZrO_2_ IL FeFET has suffered more damage than in the HfO_2_ IL FeFET. This difference in the degree of damage to the poly-Si cannot be explained by differences in the doping concentration or phase composition of the ferroelectric HZO layer but is more plausibly attributed to mechanical stress caused by the CTE difference between the ILs. Since the CTE of ZrO_2_ is more than 40% larger than that of HfO_2_, the strain stress in the ZrO_2_ IL is greater, resulting in more damage to the poly-Si channel and, thus, higher noise levels.

**Figure SN1**. (a) *S*_ID_/*I*_D_² versus frequency of the FeFETs with different ILs with *V*_DS_ fixed at 0.1 V. (b) *S*_ID_/*I*_D_² versus *V*_GS_ for FeFETs with different ILs

**Supplementary Note 2: LFN characteristics of SB FETs**

The LFN characteristics of the SB FETs are influenced by the carrier number fluctuation (CNF) at the channel and the BHF at the metal-semiconductor contact. While the CNF is generated by the carrier trapping/detrapping process from/to the defects inside the gate dielectrics, the BHF occurs mainly due to the charger fluctuation at the SB. Accordingly, the *S*_ID_/*I*_D_^2^ can be written as

$\frac{S_{\mathrm{ID}}}{{I_{D}}^{2}}={(1-\eta)}^{2}{(\frac{ln10}{S})}^{2}\frac{q^{2}N_{\mathrm{ot}}}{{C_{ox}}^{2}WLf}+A\eta^{2}{(\frac{q}{kT})}^{2}\frac{1}{{(1-e^{-q\eta\frac{V_{\mathrm{DS}}}{kT}})}^{2}}\frac{1}{f}$ .

The first term represents the contribution of CNF, while the second term accounts for contact resistance noise. In general, as shown in Figure S5, the 1/*f* noise is determined by the CNF in the low *I*_D_ region. With an increase in *V*_GS_, the contribution of the contact resistance increases, and thus the 1/*f* noise is determined by the BHF at the SB contact. During this transition phase, the *S*_ID_/*I*_D_^2^ increases with *V*_GS_ (*I*_D_) from 0.8 to 1.0 V, as shown in Figure S5. In the MFMIS FeFETs in this study exhibit the increase in the *S*_ID_/*I*_D_^2^ with *I*_D_ (Figure 3), demonstrating the transition of noise source from CNF to BHF.

**Reference**

[S1] G. Ghibaudo, O. Roux, C. Nguyen‐Duc, F. Balestra, J. Brini, Phys. Status Solidi A 1991, 124, 571.

[S2] C. Wei, Y. Z. Xiong, X. Zhou, N. Singh, S. C. Rustagi, G. Q. Lo, D. L. Kwong, IEEE Electron Device Lett 2009, 30, 668.

[S3] C. G. Theodorou, E. G. Ioannidis, F. Andrieu, T. Poiroux, O. Faynot, C. A. Dimitriadis, G. Ghibaudo, IEEE Trans. Electron Devices 2014, 61, 1161.

[S4] N. Clement, G. Larrieu, E. Dubois, IEEE Trans. Electron Devices 2011, 59, 180.

[S5] R. T. Doria, R. Trevisoli, M. de Souza, M. A. Pavanello, Solid State Electron 2014, 96, 22.

[S6] S. H. Lee, C. K. Baek, S. Park, D. W. Kim, D. K. Sohn, J. S. Lee, Y. H. Jeong, IEEE Electron Device Lett 2012, 33, 1348.

[S7] Y. Liu, S. T. Cai, C. Y. Han, Y. Y. Chen, L. Wang, X. M. Xiong, R. Chen, IEEE J. Electron Devices Soc 2019, 7, 203.

[S8] J. C. Park, S. W. Kim, C. J. Kim, S. Kim, D. H. Kim, I. T. Cho, H. I. Kwon, Appl. Phys. Lett 2010, 97, 122104.

[S9] C. J. Kim, S. Kim, D. H. Kim, I. T. Cho, H. I. Kwon, Appl. Phys. Lett 2010, 97, 122104.

[S10] T. C. Fung, G. Baek, J. Kanicki, J. Appl. Phys 2010, 108, 074518.

[S11] Y. Liu, Y. X. Huang, S. Deng, M. Wong, H. S. Kwok, R. Chen, IEEE J. Electron Devices Soc 2020, 8, 435.

[S12] W. Shin, J. Bae, J. H. Park, J. H. Lee, C. H. Kim, S. T. Lee, IEEE Electron Device Lett 2024, 45, 704.
